# Polarisation of brain dynamics in mania and depression

**DOI:** 10.1080/19585969.2026.2663032

**Published:** 2026-04-29

**Authors:** Matteo Martino, Paola Magioncalda, Benedetta Conio, Mario Amore, Zirui Huang

**Affiliations:** ^a^ Graduate Institute of Mind Brain and Consciousness, Taipei Medical University, Taipei, Taiwan; ^b^ International Master/Ph.D. Program in Medicine, College of Medicine, Taipei Medical University, Taipei, Taiwan; ^c^ Department of Neuroscience, Rehabilitation, Ophthalmology, Genetics, Maternal and Child Health, Section of Psychiatry, University of Genoa, Genoa, Italy; ^d^ IRCCS Ospedale Policlinico San Martino, Genoa, Italy; ^e^ Center for Consciousness Science, University of Michigan Medical School, Ann Arbor, MI, USA; ^f^ Department of Anesthesiology, University of Michigan Medical School, Ann Arbor, MI, USA; ^g^ Michigan Psychedelic Center, University of Michigan Medical School, Ann Arbor, MI, USA

**Keywords:** Bipolar disorder, mania, depression, brain dynamics, co-activation patterns, resting-state fMRI

## Abstract

**Introduction:**

This work investigated brain dynamics underlying manic and depressive states in bipolar disorder.

**Methods:**

Resting-state fMRI data were obtained from 69 bipolar patients—34 manic, 35 depressed*—*and 73 healthy controls. Intrinsic brain activity was modelled as a temporal sequence of discrete quasi-stationary states (co-activation patterns), which were compared between groups and related to symptomatology.

**Results:**

Mania was associated with increased occurrence of a co-activation pattern encompassing sensorimotor network areas, which positively correlated with manic symptomatology (hyperactivity and mood elevation) and manic polarity, and negatively correlated with depressive symptomatology (retardation and apathy). Depression was associated with increased occurrence of a co-activation pattern encompassing default-mode network areas, negatively correlating with manic symptomatology (hyperactivity and mood elevation).

**Conclusions:**

These findings suggest that polarisation of brain dynamics towards low-order sensorimotor/insular systems—subserving perception and modulation of the outer and inner/body environments—may reshape subjective experience and behaviour promoting immediate interaction with the environment, manifesting as mania. Conversely, polarisation towards high-order associative systems—subserving stimulus-independent associative processing—may reshape experience and behaviour favouring detachment from the environment, manifesting as depression. This framework illustrates how changes in the functional brain architecture may alter the structure of phenomenal experience and behaviour in physiology and psychopathology.

## Introduction

Bipolar disorder (BD) is a major psychiatric disorder clinically characterised by recurrent episodes of mania and depression (A.P.A 2013). Mania features elevated or irritable mood, hyperactivity/increased energy levels, logorrhoea, impulsivity, distractibility, and flight of ideas (A.P.A 2013). Conversely, depression features depressed mood, apathy, anhedonia, reduced energy, motor retardation or agitation, indecisiveness, and ruminative thinking (A.P.A 2013). These heterogeneous symptom constellations may arise from an underlying common ground and thus be grouped into specific, unitary patterns of altered subjective experience and behaviour manifesting in the manic and depressive states. However, a clear understanding of this hidden basis is still lacking.

Nevertheless, a close relationship of the manic and depressive states with alterations in the functional architecture of intrinsic brain activity can be assumed, as changes in the structure of phenomenal experience and behavioural patterns might be traced back to changes in the baseline configuration of brain activity (Martino and Magioncalda 2024a).

As previously reported (Martino and Magioncalda 2024a, 2024b; Chen et al. 2025), intrinsic brain activity is organised into large-scale functional networks unfolded along a hierarchical axis that spans spatial and temporal domains, reflecting graded connectivity patterns and corresponding differences in cortical input–output processing (Amaral and Strick 2012; Margulies et al. 2016; Gong and Zuo 2023). At the lower end of this hierarchy, sensorimotor cortices maintain direct connections with peripheral receptors—including visual, auditory, somatosensory, and interoceptive systems—enabling the detection of specific subsets of changes in the external and internal environments, as well as with peripheral effectors—namely somatomotor and autonomic systems (Craig 2002; Amaral and Strick 2012; Amaral 2012; Martino and Magioncalda 2024a; Chen et al. 2025). Intrinsic activity in these regions is embedded within the visual and somatomotor networks (VN and SMN), encompassing occipital cortex (visual areas), superior temporal gyrus (auditory areas), postcentral gyrus (somatosensory areas), precentral gyrus (motor areas), and posterior insular cortex (interoceptive areas) (Biswal et al. 1995; Yeo et al. 2011; Martino and Magioncalda 2024a; Chen et al. 2025). These networks predominantly display a local organisational profile and maximal activity at faster frequencies, consistent with their role in the local processing of inputs and outputs (Margulies et al. 2016; Wang et al. 2019; Gong and Zuo 2023; Martino and Magioncalda 2024a; Chen et al. 2025). Progressing along the hierarchy, associative cortices exhibit reciprocal connections with sensorimotor regions and a convergent organisational structure, whereby connections become increasingly complex, multimodal, and spatially distant from sensorimotor areas (Singer 1999; Amaral and Strick 2012; Martino and Magioncalda 2024a; Chen et al. 2025). Intrinsic activity in these regions is organised ventrally within the salience network (SN), encompassing anterior insula and dorsal anterior cingulate cortex, and the limbic network (LN), encompassing temporal poles and orbitofrontal cortex; and dorsally within the dorsal attention network (DAN), encompassing intraparietal sulcus and frontal eye fields, and the frontoparietal network (FPN), encompassing posterior parietal cortex and dorsolateral prefrontal cortex (Fox et al. 2006; Seeley et al. 2007; Dosenbach et al. 2008; Yeo et al. 2011; Margulies et al. 2016). These networks exhibit a distributed organisational profile and maximal activity across a broad frequency range, consistent with associative integration and top-down control (Dosenbach et al. 2008; Yeo et al. 2011; Margulies et al. 2016; Wang et al. 2019; Gong and Zuo 2023; Gong and Zuo 2025). At the upper end of this hierarchy—corresponding to the transmodal pole of the sensorimotor-to-transmodal gradient of cortical organisation—intrinsic activity is embedded within the default-mode network (DMN), encompassing anterior cingulate cortex/medial prefrontal cortex, posterior cingulate cortex, and middle temporal gyrus/temporoparietal junction (Raichle et al. 2001; Buckner et al. 2008; Yeo et al. 2011; Martino and Magioncalda 2024a; Chen et al. 2025). This network displays delayed developmental trajectories, maximal distance from sensorimotor areas along the cortical surface, a distributed and interdigitated organisational profile, and maximal activity at slower frequencies; together, these features are consistent with its associative role in transmodal integration across sensorimotor systems (Yeo et al. 2011; Margulies et al. 2016; Wang et al. 2019; Dong et al. 2021; Gong and Zuo 2023; Martino and Magioncalda 2024a; Chen et al. 2025).

Accordingly, activity within the primary/secondary sensorimotor areas subserves exteroception (visual, auditory, and somatosensory perception) and motor functions, supporting the perception of and interaction with the external environment (Amaral 2012; Martino and Magioncalda 2024a; Chen et al. 2025). Analogously, activity in the insular areas—which contains interoceptive regions—subserves interoception (including pain and pleasure) and autonomic regulation, supporting the perception and modulation of the internal bodily environment—functions closely related to affective processes (Craig 2002; Martino and Magioncalda 2024a; Chen et al. 2025). At the opposite end of the hierarchy, activity in associative areas supports thought functions that are maximally decoupled from immediate environmental input and output—i.e., associative processing and manipulation of imagery and ideas independent of environmental changes (Raichle et al. 2001; Bar et al. 2007; Buckner et al. 2008; Amaral and Strick 2012; Olson and Colby 2012; Martino and Magioncalda 2024a; Chen et al. 2025)—mediating perceptually decoupled processing of memory-related and abstract representations (complex, multimodal, and relatively invariant to specific sensory processing) (Bar et al. 2007; Smallwood 2013; Margulies et al. 2016; Murphy et al. 2018).

Evidence from resting-state functional magnetic resonance imaging (fMRI) studies has shown various alterations in the architecture of intrinsic brain activity in the manic and depressive states of BD (Öngür et al. 2010; Magioncalda et al. 2015; Martino et al. 2016a, 2016b; Brady et al. 2016; Brady et al. 2017; Zhang et al. 2019; Russo et al. 2020; Martino et al. 2020; Rey et al. 2021; Martino and Magioncalda 2022). A previous study from our group was the first to suggest that a functional reconfiguration of intrinsic brain activity characterised by an opposite imbalance between the SMN and DMN is at the basis of mania and depression (Martino et al. 2016). Various studies from our group and others have identified alterations across different fMRI metrics (including functional connectivity, temporal variability of signal amplitude, regional homogeneity, degree of centrality, and global signal representation) that are coherent with this construct: these alterations mainly consist of increases in SMN areas and/or decreases in DMN areas during mania, and decreases in SMN areas and/or increases in DMN areas during depression (Öngür et al. 2010; Magioncalda et al. 2015; Martino et al. 2016a, 2016b; Zhang et al. 2019, 2020; Martino and Magioncalda 2022; Mavar et al. 2025; Russo et al. 2020). Based on these data, we hypothesised that a tilting of network balance towards the SMN underlies manic symptomatology, while a network tilting towards the DMN underlies depressive symptomatology (Magioncalda and Martino 2022; Martino and Magioncalda 2022). However, the functional role of these brain metrics remains controversial, and interpreting their alterations in psychopathological states is challenging. Thus, how changes in intrinsic brain activity translate into alterations in the structure of subjective experience and behaviour or psychiatric symptoms remains an unanswered question.

The analysis of dynamic brain activity in BD may partially fill this knowledge gap. Brain dynamics can be characterised through a sequence of discrete (quasi-stationary) brain states, i.e., transient, momentary co-activation patterns (CAPs) in resting-state fMRI signals (Liu et al. 2013; Liu et al. 2018), which are evolutionarily conserved across mammalian species (Gutierrez-Barragan et al. 2024). Unlike approaches that infer dynamics from time-varying correlations computed over sliding windows, CAP analysis directly captures instantaneous, large-scale activation configurations at the level of individual fMRI volumes, providing an event-based description of brain dynamics with minimal assumptions about temporal stationarity. This framework enables dynamic brain organisation to be quantified in terms of the prevalence and redistribution of discrete brain states, rather than changes in averaged interregional coupling. The brain engages in a continuous exploration of the repertoire of such brain states, and each state, depending on its specific configuration across sensorimotor and associative areas, may favour a distinct pattern of input/output processing. Thus, changes in brain dynamics, such as an expansion or contraction of specific brain states, may correspondingly reshape the structure of phenomenal experience and behaviour (Huang et al. 2020; Dai et al. 2023; Huang 2023; Mawla et al. 2023). However, alterations in brain dynamics during mania and depression remain largely unexplored.

In this study, we analysed fMRI brain activity patterns in BD patients and healthy subjects. We identified distinct brain states and compared their occurrence between groups to understand how dynamic brain activity relates to manic and depressed states. We demonstrated an increased occurrence of brain states involving SMN/VN areas in mania, while an increased occurrence of brain states involving DMN areas in depression.

## Material and methods

### Clinical and MRI data

The study was conducted on 69 BD patients, assessed during manic state (*n* = 34) or depressive state (*n* = 35), and 73 healthy controls (HC). All participants underwent comprehensive clinical assessment and MRI. Demographic and clinical characteristics of the sample are reported in Supplementary Table 1.

Inclusion criteria for patients were: diagnosis of BD according to DSM criteria (A.P.A 2013); current manic or depressive episode established through clinical evaluation and DSM criteria in an inpatient setting; Young Mania Rating Scale (YMRS) score ≥13 for manic patients or 17-item Hamilton Depression Rating Scale (HAM-D) score ≥18 for depressed patients to confirm episode severity; age between 18 and 60 years; and ability to provide written informed consent. Exclusion criteria included: diagnosis of other major neuropsychiatric disorders (e.g., schizophrenia, intellectual disability, or neurocognitive disorders); neurological diseases (e.g., stroke, cerebrovascular malformations, or epilepsy), history of head injury with loss of consciousness lasting ≥5 min, or severe or chronic medical illnesses; current alcohol or substance abuse within the previous 3 months or a lifetime history of alcohol or substance addiction; previous treatment with electroconvulsive therapy, chemotherapy, or brain radiotherapy; left-handedness; pregnancy or lactation; and contraindications to MRI (e.g., claustrophobia or metal implants). HC did not meet DSM criteria for any psychiatric disorder, either current or past, had HAM-D and YMRS scores <8, and met the same exclusion criteria applied to patients.

Detailed information on the sample, including clinical and MRI data collection, is provided in the Supplementary Material.

### fMRI data processing

Functional MRI data were preprocessed using AFNI. After discarding initial frames, slice timing correction and rigid-body motion correction were applied, with frames exceeding 0.4 mm of framewise displacement being censored. Functional images were coregistered to anatomical images and normalised to Talairach space. Data were then band-pass filtered (0.01–0.1 Hz) and nuisance regression was performed to remove signal components related to drift, motion, and white matter/cerebrospinal fluid signals. Spatial smoothing with a 6-mm Gaussian kernel and intensity normalisation was then applied. Global signal regression was not performed to avoid potential biases in characterising anticorrelations (Huang et al. 2020).

### Co-activation pattern analysis

CAPs were identified using a k-means clustering algorithm implemented in MATLAB R2023b. To reduce computational complexity, fMRI data from HC were preprocessed by z-normalizing, resampling to 6 × 6 × 6 mm voxels, and masking with a grey matter mask, resulting in 6088 voxels per frame. Frames censored during preprocessing were excluded from the analysis. All remaining frames were then concatenated and clustered using the k-means algorithm. While the optimal number of clusters (k) for CAP identification remains an active area of research, previous studies suggest that a range of 5 to 8 CAPs provides a balance between explained variance and generalisability, demonstrating robust inter- and intra-subject consistency in humans (Zhang et al. 2020; Chen et al. 2023), cross-species validity (Gutierrez-Barragan et al. 2024), and sensitivity to disease-related alterations (Huang et al. 2020; Mawla et al. 2023). Accordingly, analyses were conducted with *k* = 6 and *k* = 8 to identify the most informative solution for characterising CAPs. For each cluster, a representative CAP map was generated by averaging and z-normalizing the corresponding frames. CAPs were named based on their resemblance to major canonical networks. CAPs in patients with BD were labelled by applying the centroids derived from the HC analysis to each patient’s data using a supervised machine learning approach based on maximum spatial similarity. Importantly, HC and BD data were not combined for the initial clustering to avoid biasing the CAP definitions with potential disease-related effects. See Supplementary Figure 1.

To quantify the prevalence of each CAP, the occurrence rate was calculated for each fMRI scan. This was done by dividing the number of frames assigned to a specific CAP by the total number of frames in the scan. For instance, an occurrence rate of 0.3 for a given CAP indicates that this pattern was present 30% of the time during the scan. This readily interpretable metric facilitates comparisons of CAP prevalence across individuals.

### Statistical analysis

Differences in CAP occurrence rates between HC, mania, and depression were investigated. Each CAP’s occurrence rate was entered into an ANOVA (with age and sex as covariates), followed by pairwise post-hoc comparisons (with Bonferroni correction).

The relationship between the occurrence rates of CAPs and manic-depressive symptomatology was examined using Spearman’s correlation analysis within the BD group. Specifically, manic and depressive symptoms were quantified using the YMRS and HAM-D. To capture manic-depressive psychopathology along a dimensional continuum encompassing manic, depressive, and mixed features, a YMRS/HAM-D ratio was calculated as the difference between mean YMRS and HAM-D item scores $\left(\frac{\text{YMRS total score}}{\text{YMRS number of items}}-\frac{\text{HAMD total score}}{\text{HAMD number of items}}\right)$, with positive values indicating predominance of manic symptomatology and negative values indicating predominance of depressive symptomatology. Correlations between YMRS/HAM-D ratio and CAPs were examined first and corrected for multiple comparisons using Bonferroni correction across CAPs. Associations with YMRS and HAM-D total scores were examined next, with correction applied across CAPs. Moreover, exploratory symptom-level analyses were conducted to identify specific manic or depressive symptoms potentially contributing to the observed associations; these analyses were restricted to CAPs significantly associated with YMRS/HAM-D scores and corrected within each CAP and scale.

Finally, the relationship between the occurrence rates of CAPs and other clinical variables—including duration of the current episode, duration of illness, lifetime number of episodes, and the dominant polarity (manic or depressive)—was examined using Spearman’s correlation analysis within the BD group. Manic-depressive polarity was quantified using an episode-based polarity ratio $\left(\frac{1+\text{ N manic episodes}+\text{N hypomanic episodes​ }+0.5\times \text{N mixed episodes​​}}{1+\text{ N depressive episodes​ }+0.5\times \text{N mixed episodes​​}}\right)$,providing a continuous measure of longitudinal polarity balance (with a constant added to avoid zero values, and mixed episodes included in both terms to reflect their dual valence): values ≈1 indicate a balanced lifetime polarity, values >1 a predominance of manic/hypomanic episodes, and values <1 a predominance of depressive episodes.

## Results

### Co-activation patterns of brain activity

Brain dynamics were investigated by identifying and extracting CAPs of brain activity. CAPs were classified according to the main intrinsic brain networks, based on visual inspection and by quantifying their alignment with predefined networks (Yeo et al. 2011). We found that *k* = 6 represented the minimum value to clearly discriminate the canonical networks in the spatial maps of the CAPs and provided the most informative solution for characterising CAPs in patients with BD.

**Figure 1.**
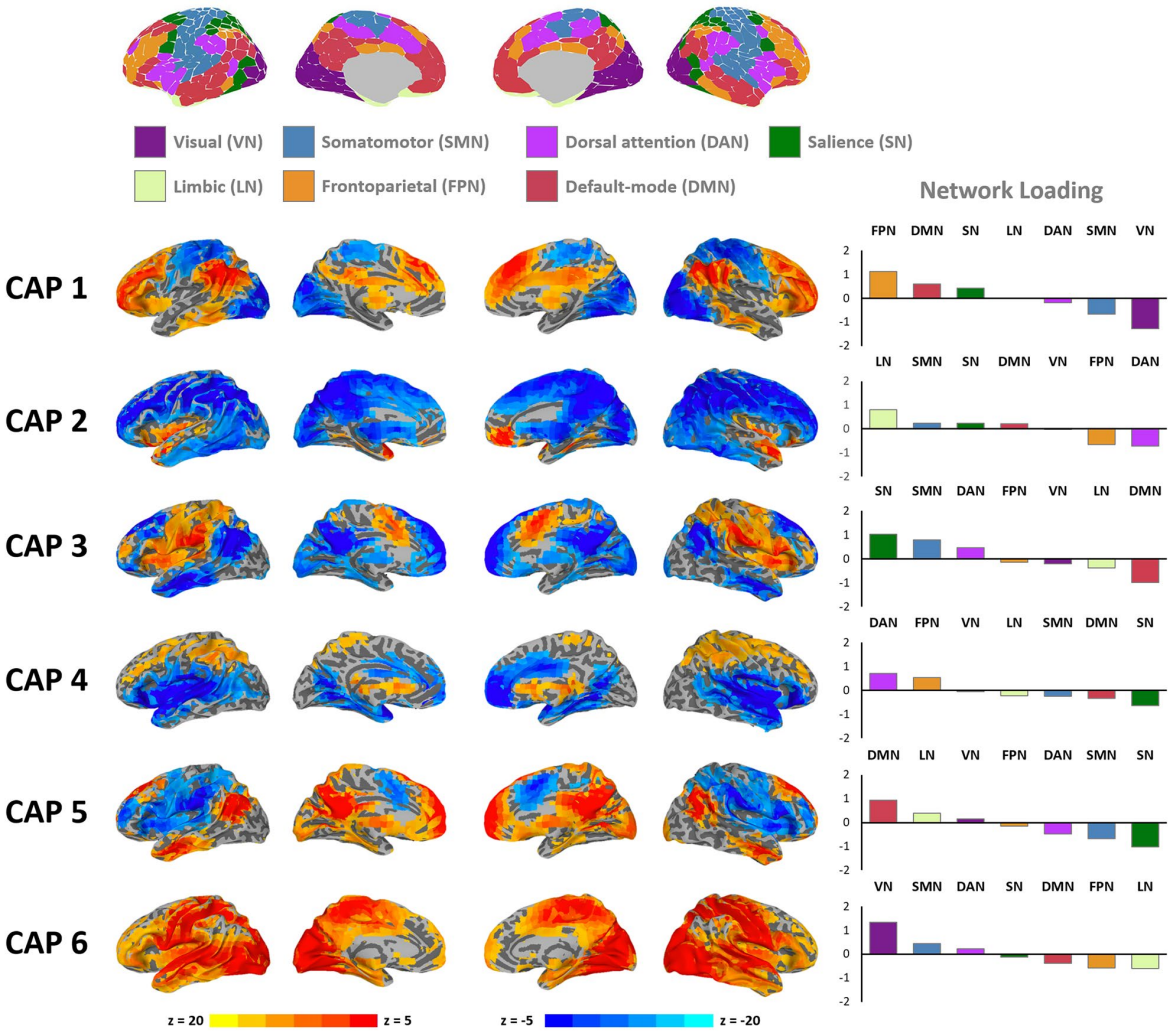
Spatial maps and network contributions of CAPs (*k* = 6). Co-activation pattern (CAP) maps reveal distinct spatial configurations of coordinated brain activity. The accompanying bar plots quantify the involvement of key brain networks—visual (VN), somatomotor (SMN), salience (SN), dorsal attention (DAN), limbic (LN), frontoparietal (FPN), and default-mode (DMN)—in each CAP.

Specifically, CAP 1 is dominated by the co-activation of regions primarily belonging to the FPN, along with de-activation of regions mainly belonging to the SMN and VN. CAP 2 involves the co-activation of regions primarily belonging to the LN, as well as de-activation of the remaining areas, mainly encompassing the DAN. CAP 3 is dominated by the co-activation of regions primarily belonging to the SN, along with de-activation of regions mainly included in the DMN. CAP 4 involves the co-activation of dorsal regions mainly encompassing the DAN, as well as de-activation of ventral regions mainly encompassing the SN. CAP 5 is dominated by the co-activation of regions primarily belonging to the DMN, along with de-activation of areas primarily belonging to the SN. Finally, CAP 6 is dominated by the co-activation of regions primarily belonging to the SMN and VN. See Figure 1.

### Co-activation patterns of brain activity in mania and depression

Differences in occurrence rates across the six CAPs were investigated between HC, mania, and depression. Brain dynamics showed a balanced distribution of occurrence rates across CAPs in HC. In contrast, brain dynamics appeared unbalanced in the manic and depressive states of BD.

In mania, brain dynamics were dominated by CAP 6 (SMN+VN+), which showed a statistically significant increase in occurrence rates in manic patients compared with depressed patients (Bonferroni-corrected *p* < 0.05) and HC (uncorrected *p* < 0.05). Additionally, in manic patients, CAP 4 (DAN+) showed a trend towards decreased occurrence compared with HC (*p* < 0.1). CAP 1 (FPN+), CAP 2 (LN+), CAP 3 (SN+), and CAP 5 (DMN+) did not exhibit clear changes.

In depression, brain dynamics were dominated by CAP 5 (DMN+), which showed a statistically significant increase in occurrence rates in depressed patients compared with manic patients and HC (Bonferroni-corrected *p* < 0.05). Additionally, in depressed patients, CAP 3 (SN+) showed a trend towards increased occurrence compared with manic patients and HC, whereas CAP 2 (LN+) showed a trend towards decreased occurrence compared with manic patients and HC, and CAP 4 (DAN+) a trend towards decreased occurrence compared with HC (all *p* < 0.1). CAP 1 (FPN+) and CAP 6 (SMN+VN+) did not exhibit clear changes.

**Figure 2.**
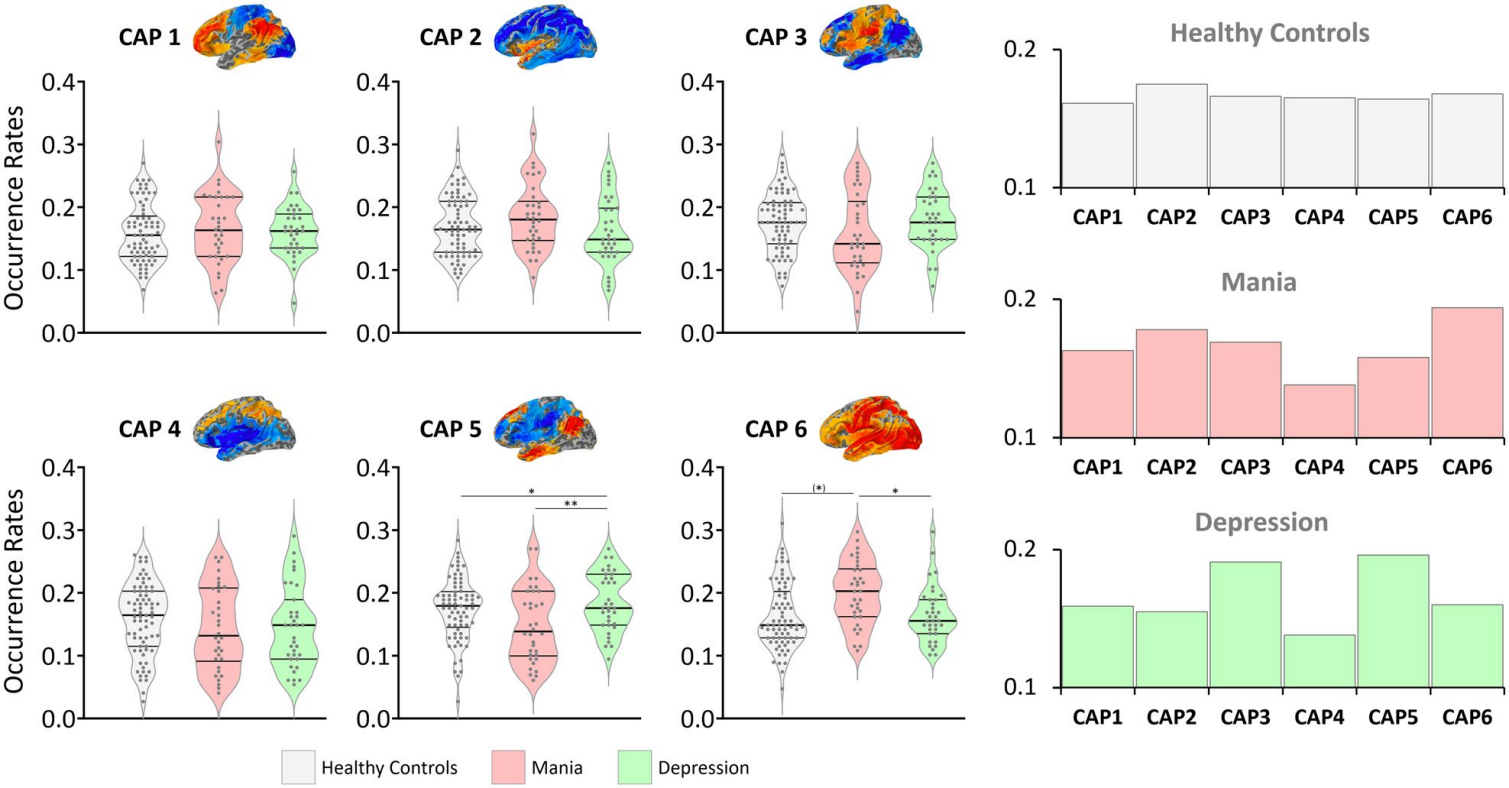
Differences in CAP occurrence rates between healthy controls, mania, and depression (*k* = 6). Differences in occurrence rates of co-activation patterns (CAPs) between healthy controls, mania, and depression were assessed using ANOVA, with age and sex as covariates, followed by post-hoc comparisons. (*) *p* < 0.05 (uncorrected); * *p* < 0.05 (Bonferroni-corrected); ** *p* < 0.01 (Bonferroni-corrected). Bar plots show the average occurrence of each CAP, adjusted for age and sex.

See Figure 2 and Table 1.

**Table 1.** Differences in CAP occurrence rates between healthy controls, mania, and depression (*k* = 6).

	**HC**	**M**	**D**	**ANOVA**	**M vs. HC**	**D vs. HC**	**M vs. D**
**CAP**	*Mean (SD)*	*Mean (SD)*	*Mean (SD)*	*F (p)*	p (p*)	p (p*)	p (p*)
**CAP 1**	0.161 (0.04)	0.163 (0.05)	0.159 (0.03)	0.05 (0.94)	M > HC 0.89	D < HC 0.84	D < M 0.74
**CAP 2**	0.175 (0.04)	0.178 (0.05)	0.155 (0.05)	2.41 (0.09)	M > HC 0.82	D < HC 0.067	D < M 0.050
**CAP 3**	0.166 (0.04)	0.169 (0.06)	0.191 (0.04)	2.88 (0.06)	M > HC 0.84	D > HC 0.028	D > M 0.056
**CAP 4**	0.165 (0.05)	0.138 (0.06)	0.138 (0.06)	2.56 (0.08)	M < HC 0.057	D < HC 0.054	M-D 0.99
**CAP 5**	0.164 (0.04)	0.158 (0.05)	0.196 (0.04)	**6.32 (0.002)**	M < HC 0.61	**D > HC 0.004 (0.013)**	**D > M 0.001 (0.004)**
**CAP 6**	0.168 (0.05)	0.194 (0.05)	0.160 (0.04)	**4.42 (0.014)**	**M > HC 0.024** (0.07)	D < HC 0.52	**M > D 0.006 (0.017)**

Differences in occurrence rates of co-activation patterns (CAPs) between healthy controls (HC), mania (M), and depression (D) were assessed using ANOVA, with age and sex as covariates, followed by post-hoc comparisons (values are adjusted for age and sex). *p* = uncorrected post-hoc contrasts; p* = Bonferroni-corrected post-hoc contrasts. Values in bold indicate significant results.

### Relationship of co-activation patterns with clinical features

Relationships between occurrence rates of the six CAPs and manic-depressive symptomatology were investigated in BD. The occurrence rates of CAP 5 (DMN+) negatively correlated with the YMRS/HAM-D ratio and YMRS total score; conversely, the occurrence rates of CAP 6 (SMN+VN+) positively correlated with the YMRS/HAM-D ratio and YMRS total score, and negatively correlated with HAM-D total score (all Bonferroni-corrected *p* < 0.05). No significant correlations were observed for the remaining CAPs. At the symptom-level exploration of CAPs significantly associated with YMRS/HAM-D scores, CAP 5 (DMN+) occurrence negatively correlated with YMRS item 1 (elevated mood) and item 2 (increased motor activity/energy levels); conversely, CAP 6 (SMN+VN+) occurrence positively correlated with YMRS item 1 (elevated mood) and item 2 (increased motor activity/energy levels), and negatively correlated with HAM-D item 7 (work and activities) and item 8 (retardation) (all Bonferroni-corrected *p* < 0.05).

Finally, among other clinical features, CAP 1 (FPN+) and CAP 6 (SMN+VN+) occurrence positively correlated with manic-depressive polarity, reflecting predominance of manic episodes (Bonferroni-corrected *p* < 0.05), with no other significant correlations observed.

**Figure 3.**
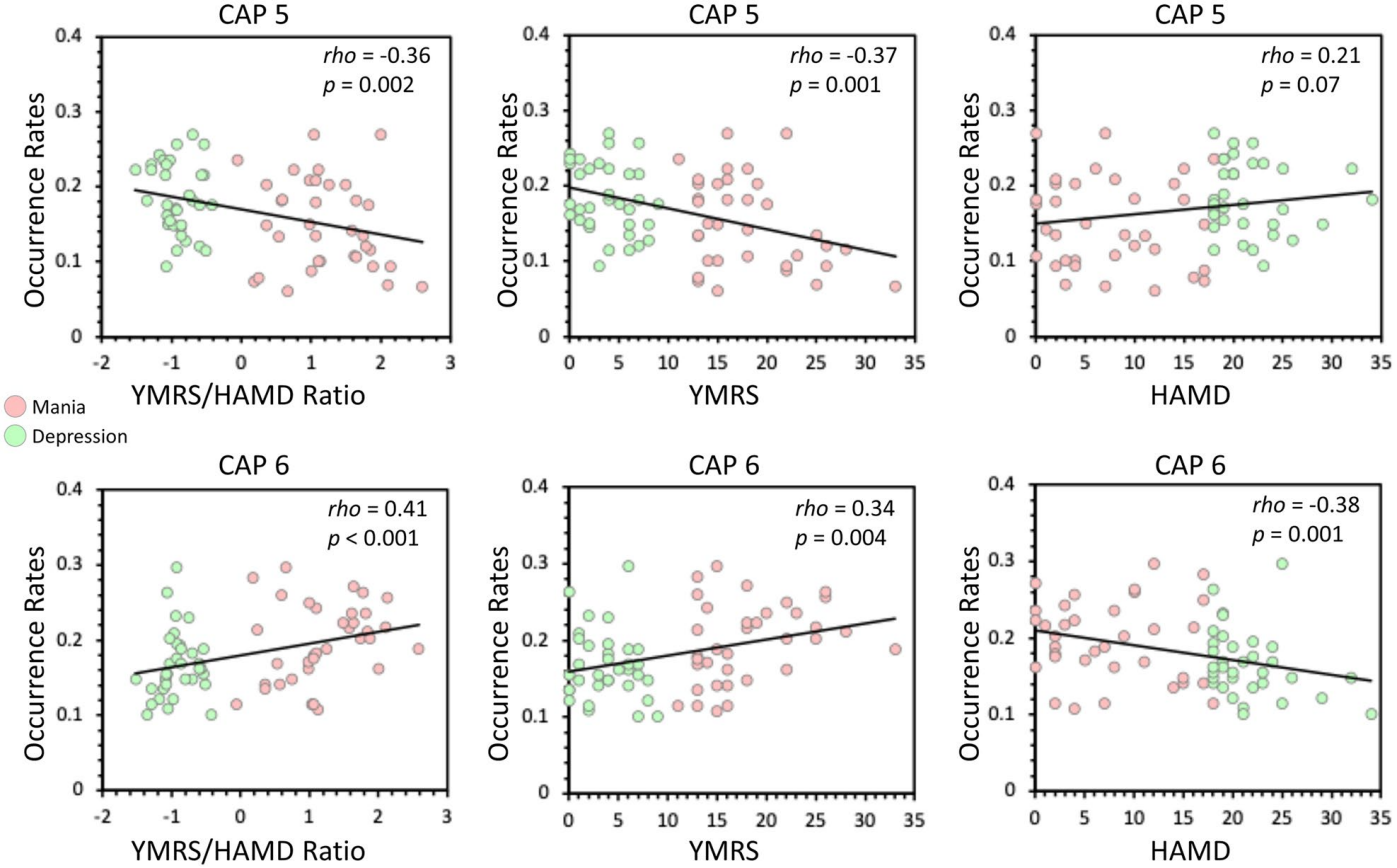
Relationship of CAP occurrence rates with manic-depressive symptomatology (*k* = 6). The relationship between occurrence rates of co-activation patterns (CAPs) and manic-depressive symptomatology—including the Young Mania Rating Scale (YMRS)/Hamilton Depression Rating Scale (HAM-D) ratio, YMRS total score, and HAM-D total score—was assessed using Spearman correlation analysis. Significant results survived Bonferroni correction.

See Figure 3, Table 2, and Supplementary Table 3a.

**Table 2.** Relationship of CAP occurrence rates with manic-depressive symptomatology (*k* = 6).

	**CAP 5**	**CAP 6**
**MANIC-DEPRESSIVE SYMPTOMATOLOGY**	*rho (p)*	*rho (p)*
**YMRS/HAM-D RATIO**	**−0.36 (0.002)**	**0.41 (<0.001)**
**YMRS TOTAL SCORE**	**−0.37 (0.001)**	**0.34 (0.004)**
YMRS 1 (elevated mood)	**−0.37 (0.001)**	**0.42 (<0.001)**
YMRS 2 (increased motor activity/energy levels)	**−0.39 (0.001)**	**0.42 (<0.001)**
YMRS 3 (sexual interest)	−0.17 (0.14)	0.2 (0.10)
YMRS 4 (sleep)	−0.22 (0.06)	*0.25 (0.03)*
YMRS 5 (irritability)	−0.17 (0.16)	0.05 (0.64)
YMRS 6 (speech - rate and amount)	*−0.25 (0.032)*	*0.31 (0.008)*
YMRS 7 (language/thought disorder)	−0.19 (0.11)	0.09 (0.45)
YMRS 8 (content)	*−0.30 (0.012)*	0.22 (0.06)
YMRS 9 (disruptive/aggressive behaviour)	*−0.29 (0.013)*	0.19 (0.11)
YMRS 10 (appearance)	−0.18 (0.13)	0.23 (0.049)
YMRS 11 (insight)	−0.13 (0.27)	−0.01 (0.87)
**HAM-D TOTAL SCORE**	0.21 (0.07)	**−0.38 (0.001)**
HAM-D 1 (depressed mood)	0.19 (0.11)	*−0.24 (0.040)*
HAM-D 2 (feelings of guilt)	0.13 (0.28)	*−0.24 (0.040)*
HAM-D 3 (suicide)	0.15 (0.19)	*−0.33 (0.005)*
HAM-D 4 (insomnia: early in the night)	−0.01 (0.95)	−0.15 (0.18)
HAM-D 5 (insomnia: middle of the night)	−0.06 (0.58)	−0.06 (0.62)
HAM-D 6 (insomnia: early hours of the morning)	−0.15 (0.21)	−0.18 (0.12)
HAM-D 7 (reduced work and activities)	*0.28 (0.019)*	**−0.38 (0.001)**
HAM-D 8 (retardation)	*0.25 (0.037)*	**−0.35 (0.002)**
HAM-D 9 (agitation)	−0.18 (0.13)	0.19 (0.10)
HAM-D 10 (anxiety psychic)	−0.12 (0.32)	−0.13 (0.25)
HAM-D 11 (anxiety somatic)	0.08 (0.48)	−0.02 (0.81)
HAM-D 12 (somatic symptoms gastro-intestinal)	0.10 (0.37)	−0.17 (0.14)
HAM-D 13 (general somatic symptoms)	0.12 (0.30)	−0.13 (0.27)
HAM-D 14 (genital symptoms)	0.01 (0.95)	−0.14 (0.24)
HAM-D 15 (hypochondriasis)	0.07 (0.54)	−0.05 (0.63)
HAM-D 16 (loss of weight)	0.04 (0.71)	−0.23 (0.05)
HAM-D 17 (insight)	0.02 (0.83)	−0.10 (0.38)

The relationship between occurrence rates of co-activation patterns (CAPs) and manic-depressive symptomatology—Young Mania Rating Scale (YMRS) and Hamilton Depression Rating Scale (HAM-D) scores—was assessed using Spearman correlation analysis. Italicised values indicate significant results; bold values indicate significant results surviving Bonferroni correction.

CAP analyses for *k* = 8, including group differences and clinical correlations, are reported in the Supplementary Material.

## Discussion

### Main findings

**Figure 4.**
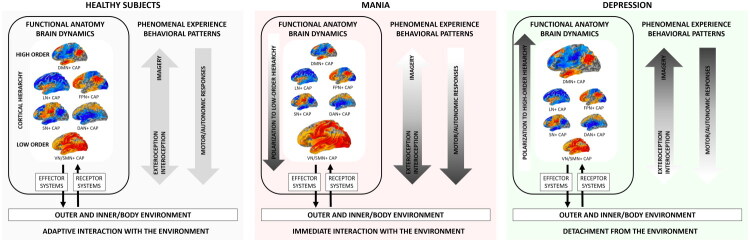
Polarisation of brain dynamics in mania and depression. Brain dynamics in healthy individuals involve a balanced repertoire of brain states organised along the sensorimotor-to-transmodal gradient in the cortical hierarchy. Lower-tier brain states—dominated by sensorimotor and insular areas, part of the VN and SMN—facilitate exteroception, interoception, and motor/autonomic responses, whereas higher-tier brain states—dominated by associative areas, part of the DMN—support perceptual-decoupled imagery. In mania, brain dynamics polarise towards low-order sensorimotor/insular states, favouring immediate interaction with the environment that may manifest in corresponding symptom patterns, such as hyperactivity/impulsivity and heightened affective responses. Conversely, in depression, brain dynamics polarise towards high-order associative states, leading to detachment from the environment that may manifest in corresponding symptom patterns, such as thought rumination and reduced motor/affective responses to external stimuli. These abnormal polarizations reflect maladaptive tuning of brain state dynamics to the environment. Abbreviations: CAP, co-activation pattern; VN, visual network; SMN, somatomotor network; SN, salience network; DAN, dorsal attention network; LN, limbic network; FPN, frontoparietal network; DMN, default-mode network.

The main findings are as follows. Mania is characterised by increased occurrence rates of a CAP involving sensorimotor and insular areas primarily encompassing the SMN and VN, which positively correlate with manic symptomatology (mainly hyperactivity and mood elevation) and manic polarity, and negatively correlate with depressive symptomatology (mainly retardation and apathy). Conversely, depression is characterised by increased occurrence rates of a CAP involving associative areas primarily encompassing the DMN, which negatively correlate with manic symptomatology (mainly hyperactivity and mood elevation). See Figure 4.

### Dynamic reconfigurations of intrinsic brain activity and manic-depressive psychopathology

This work is, to our knowledge, the first to employ CAP analysis to explore whole-brain dynamics in the manic and depressive states of BD. The present findings align with our hypothesis linking SMN dominance to mania and DMN dominance to depression (Magioncalda and Martino 2022; Martino and Magioncalda 2022). Our results substantiate previous evidence of changes in temporally-averaged metrics (Öngür et al. 2010; Magioncalda et al. 2015; Martino et al. 2016; 2016; Zhang et al. 2019, 2020; Russo et al. 2020), suggesting that their increase in SMN areas during mania reflects a higher occurrence of SMN-dominated brain states, while their increase in DMN areas during depression reflects a higher occurrence of DMN-dominated brain states. Our findings are also consistent with the only prior CAP study in (hypo)mania and depression, which specifically examined amygdala connectivity dynamics (Rey et al. 2021). That study found that amygdala predominantly engaged with a sensorimotor-interoceptive CAP during manic states and with a DMN-dominated CAP during depressive states (Rey et al. 2021). Going beyond this region-specific approach, our research offers an original contribution by mapping the broader dynamic architecture of intrinsic cortical activity across the whole brain. This whole-brain perspective allows us to generalise and expand upon previous findings, offering a foundational understanding of the functional reconfigurations of intrinsic brain activity associated with manic-depressive psychopathology. Importantly, our results provide stronger empirical support for, and further refinement of, the theoretical framework we previously proposed (Magioncalda and Martino 2022; Martino and Magioncalda 2022; Martino and Magioncalda 2024a), reducing the exploratory nature of these findings and enhancing their interpretability.

In particular, in mania, the brain tends to explore a brain state composed of the sensorimotor and posterior insular cortices relatively more frequently, particularly compared with depression. These structures lie at the low level of the cortical hierarchy and are directly connected with the outer and inner/body environment *via* receptor (exteroceptor/interoceptor) systems and effector (somatomotor/autonomic) systems (Craig 2002; Amaral 2012; Margulies et al. 2016). Hence, a polarisation of the repertoire of brain dynamics towards this state may enhance the detection of stimuli from the outer and inner/body environment, favouring the dominance of exteroceptive and interoceptive elements (over imagery) in phenomenal experience, along with the associated motor and autonomic responses (Martino and Magioncalda 2024a). Such change in the structure of phenomenal experience and behavioural pattern may promote immediate interaction with the outer and inner environment, manifesting in typical manic symptomatology, such as hyperactivity/impulsive behaviour and heightened affective responses (Martino and Magioncalda 2022; Martino and Magioncalda 2024a). This clinical relationship is further supported by our correlation results. Accordingly, we suggest that an over-tuning of intrinsic brain activity and related phenomenal experience/behavioural patterns onto the current environment represents the core of mania.

Conversely, in depression, the brain tends to explore a brain state composed of the associative cortices relatively more frequently, particularly compared with mania. These areas lie at the high level of the cortical hierarchy and are reciprocally connected with the sensorimotor/insular areas (but not with the external and internal/body environment) (Buckner et al. 2008; Amaral and Strick 2012; Margulies et al. 2016). Hence, a polarisation of the repertoire of brain dynamics towards this state may favour associative processing independent of environmental stimuli, promoting the dominance of imagery (over perceptual elements) in phenomenal experience, while reducing behavioural responses (particularly to environmental stimuli) (Martino and Magioncalda 2024a). Such change in the structure of phenomenal experience and behavioural pattern may favour detachment from the environment, manifesting in typical depressive symptomatology, such as thought ruminations and reduced motor/affective responses (particularly to environmental stimuli) (Martino and Magioncalda 2022; Martino and Magioncalda 2024a). This clinical relationship could not be directly tested in the present study, as the clinical scales used do not assess rumination or distinguish between imagery- and perceptually related motor/affective responses. However, DMN activation has been robustly associated with thought rumination (Zhou et al. 2020). Accordingly, we suggest that a de-tuning of intrinsic brain activity and related phenomenal experience/behavioural patterns from the environment represents the core of depression.

A discussion of study limitations and future directions is provided in the Supplementary Material.

## Conclusions

In summary, an abnormal polarisation of brain dynamics towards a brain state dominated by low-order sensorimotor/insular areas may reshape the baseline structure of subjective experience and behavioural patterns promoting immediate interaction with the environment (with a corresponding propensity for action/impulsive behaviour and affective responses), as the core of mania. Conversely, an abnormal polarisation towards a brain state dominated by high-order associative areas may reshape subjective experience and behaviour favouring detachment from the environment (with a corresponding propensity for thought rumination with inhibition of action and affective responses), as the core of depression. This construct may represent a potential common basis for integrating the heterogeneous functional brain alterations and manic-depressive symptomatology within a unitary framework, while also illustrating how changes in the functional architecture of intrinsic brain activity can alter the structure of phenomenal experience and behaviour.

## Supplementary Material

Supplemental Material

## Data Availability

The dataset generated and analysed during this study, as well as the codes used, are available from the corresponding author upon reasonable request.
