# Supplementary material for: Polarisation of brain dynamics in mania and depression
Source: Dialogues Clin Neurosci. 2026 Apr 29;28(1):10–20. doi: 10.1080/19585969.2026.2663032 (PMC13130228; doi:10.1080/19585969.2026.2663032)
Supplement: Supplemental Material [file TDCN_A_2663032_SM6464.pdf]

Supplementary Material

POLARISATION OF BRAIN DYNAMICS IN MANIA AND DEPRESSION

Matteo Martino, Paola Magioncalda, Benedetta Conio, Mario Amore, Zirui Huang

Additional methods - clinical and MRI data collection

Patients with bipolar disorder (BD) were recruited from the Psychiatric Clinic of the San Martino Polyclinic Hospital and the Department of Neuroscience at the University of Genoa (Italy). Healthy controls (HC) were recruited from the Genoa metropolitan area.
All participants underwent a comprehensive clinical assessment using standardised instruments to characterise diagnostic features, course of illness, and pharmacological treatment. These included the Mini International Neuropsychiatric Interview (MINI), the Structured Clinical Interview for DSM Axis I Disorders-Patient Edition (SCID-I/P), the Structured Interview for Mood Disorders-Revised (SIMD-R), the Young Mania Rating Scale (YMRS), and the 17-item Hamilton Depression Rating Scale (HAM-D). General medical, physiological, pathological, and psychopathological histories were collected, and a full psychiatric examination was performed.
All participants underwent magnetic resonance imaging (MRI) on a 1.5-Tesla GE Signa HDxt scanner using an 8-channel head coil. Foam pads were used to minimise head motion and reduce scanner noise. High-resolution anatomical T1-weighted images were acquired using a three-dimensional fast spoiled gradient-echo (3D-FSPGR) sequence. A total of 188 sagittal slices were collected with the following parameters: repetition time (TR) = 11.70 ms, echo time (TE) = 5.12 ms, inversion time = 500 ms, flip angle = 8°, matrix size = 256×256, slice thickness = 1 mm, and in-plane resolution = 1×1 mm^2. Resting-state functional MRI (fMRI) data were acquired in darkness, with participants instructed to keep their eyes closed, remain awake, and minimise movement. Functional images were collected using a gradient-echo echo-planar imaging (EPI) sequence sensitive to blood-oxygen-level-dependent (BOLD) contrast, with the following parameters: TR = 2000 ms, TE = 30 ms, flip angle = 90°, field of view (FOV) = 24 cm. Whole-brain volumes were acquired in 33 contiguous transverse slices, with slice thickness = 4 mm, interslice gap = 1 mm, and in-plane resolution of 3.75×3.75 mm^2. For each participant, resting-state fMRI scanning lasted 5 minutes, yielding 150 volumes. The MRI procedure was successfully completed in the sample analysed in the present study, including patients in manic or depressive states. All patients were inpatients at the time of scanning and were clinically monitored throughout the procedure; they were accompanied to the scanner by the treating psychiatrist or an assigned psychiatry resident. No sedative medication was administered specifically for MRI acquisition, in order to avoid introducing additional confounding effects on brain activity. Standard measures were used to promote cooperation and minimise motion, including clear instructions prior to scanning and continuous monitoring during image acquisition.
BD patients and/or HC included in the present study partially overlap with cohorts examined in our previous publications[1-18]. Those prior studies focused on static or temporally averaged measures of intrinsic brain activity assessed using resting-state fMRI (i.e., functional connectivity[1, 4, 12, 14, 15]; temporal variability of signal amplitude[3, 7, 10, 15, 18]; regional homogeneity and degree centrality[13]; global signal representation[11]), white matter microstructure assessed using diffusion MRI[2, 4, 6, 8], or peripheral immune and blood-based measures[6, 17]. Importantly, analyses of brain dynamics of resting-state fMRI data are presented here for the first time and have not been previously performed in these participants.

Additional results: Co-activation pattern analyses for k=8

Co-activation patterns of brain activity

An additional co-activation pattern (CAP) analysis using k=8 clusters was performed to examine network dynamics at finer granularity in HC and BD patients.

CAPs were classified according to the canonical brain networks, including visual network (VN), somatomotor network (SMN), salience network (SN), dorsal attention network (DAN), limbic network (LN), frontoparietal network (FPN), and default-mode network (DMN)[19]. This analysis identified 4 mirrored CAP pairs characterised by opposite fMRI co-activation, reflecting a cyclical fluctuation of a single brain state[20]. Specifically, CAP 1 is dominated by FPN co-activation and SMN/VN de-activation, whereas its mirror CAP 3 is dominated by SMN/VN co-activation and FPN de-activation, together forming a CAP–antiCAP of entangled FPN–SMN/VN oscillation. CAP 2 is dominated by DMN co-activation and SN de-activation, whereas its mirror CAP 4 is dominated by SN co-activation and DMN de-activation, together forming a CAP–antiCAP of entangled DMN–SN oscillation. CAP 5 involves dorsal cortex co-activation and ventral cortex de-activation, whereas its mirror CAP 7 involves ventral cortex co-activation and dorsal cortex de-activation, together forming a CAP–antiCAP of dorsal–ventral cortex oscillation. Finally, CAP 6 involves global co-activation, whereas its mirror CAP 8 involves global de-activation, together forming a CAP–antiCAP of global signal oscillation. See **Supplementary Figure 2**.

Co-activation patterns of brain activity in mania and depression

Differences in occurrence rates across the eight CAPs were investigated between HC, mania, and depression.

Brain dynamics in HC showed a balanced distribution of occurrence rates across three CAP–antiCAP pairs, namely CAP 1–CAP 3 (FPN–SMN/VN oscillation), CAP 2–CAP 4 (DMN–SN oscillation), and CAP 5–CAP 7 (dorsal–ventral cortex oscillation), while reduced occurrence rates were observed for the CAP 6–CAP 8 pair (global signal oscillation).

In depression, brain dynamics were dominated by the CAP 2–CAP 4 pair (DMN–SN oscillation), which showed a statistically significant increase in occurrence rates in depressed patients compared with manic patients and HC (Bonferroni-corrected $p<0.05$). By contrast, occurrence rates of the CAP 5–CAP 7 pair (dorsal–ventral cortex oscillation) showed a statistically significant decrease in depressed patients compared with HC (uncorrected $p<0.05$). The CAP 1–CAP 3 pair (FPN–SMN/VN oscillation) and the CAP 6–CAP 8 pair (global signal oscillation) did not exhibit clear changes.

In mania, occurrence rates of the CAP 5–CAP 7 pair (dorsal–ventral cortex oscillation) decreased compared with HC (Bonferroni-corrected $p<0.05$ for CAP 5; trend-level $p<0.1$ for CAP 7). The CAP 1–CAP 3 pair (FPN–SMN/VN oscillation), the CAP 2–CAP 4 pair (DMN–SN oscillation), and the CAP 6–CAP 8 pair (global signal oscillation) did not exhibit clear changes.

See **Supplementary Figure 3** and **Supplementary Table 2**.

Relationship of co-activation patterns with clinical features

Relationships between occurrence rates of the eight CAPs and clinical features were investigated in BD. The occurrence rates of the CAP 2–CAP 4 pair (DMN–SN oscillation) negatively correlated with the YMRS/HAM-D ratio and YMRS total score (all uncorrected $p<0.05$). Conversely, the occurrence rates of the CAP 1–CAP 3 pair (FPN–SMN/VN oscillation) positively correlated with the YMRS/HAM-D ratio, and CAP 3 occurrence negatively correlated with HAM-D total score (all uncorrected $p<0.05$). Furthermore, the occurrence rates of the CAP 1–CAP 3 pair (FPN–SMN/VN oscillation) also positively correlated with manic-depressive polarity, reflecting predominance of manic episodes (Bonferroni-corrected $p<0.05$). No other significant correlations were observed. See **Supplementary Table 3b**.

Dynamic reconfigurations of intrinsic brain activity and manic-depressive psychopathology

These results suggest that, physiologically and at rest, brain dynamics are typically characterised by a balanced exploration of three complementary brain states, each arising from entangled, counterpolar network oscillations. These include: (a) a brain state of FPN–SMN/VN oscillation, which can be situated along the dorsal component of the sensorimotor–associative axis, coupling regions primarily engaged in exteroceptive–motor processing with regions involved in associative processing; (b) a brain state of DMN–SN oscillation, which can be situated along the ventral component of the sensorimotor–associative axis, coupling regions supporting interoceptive–autonomic processing with regions involved in associative processing; and (c) a brain state of dorsal–ventral cortex oscillation, which can be situated along the dorsal–ventral axis, coupling regions engaged in exteroceptive–motor–related processing with regions engaged in interoceptive–autonomic–related processing.

In depression, brain dynamics are marked by increased exploration of the brain state dominated by DMN–SN oscillation, potentially at the expense of the brain state dominated by dorsal–ventral cortex oscillation. This polarisation of the repertoire of brain dynamics toward a state oscillating along the ventral axis of interoceptive–associative processing may promote an abnormal dominance of affective–imagery coupling with detachment from the external environment. Specifically, bodily and affective states—rather than external stimuli—may excessively drive imagery, and, in turn, imagery—rather than external stimuli—may excessively drive emotions and saliency, potentially manifesting in key depressive symptomatology (e.g., depressive thought ruminations and reduced affective/motor responses to external stimuli). Thus, integrating results from k=6 and k=8 analyses, alterations in brain dynamics converge on a core role of de-tuning of intrinsic brain activity and corresponding phenomenal/behavioural patterns from the external environment in depression.

In mania, brain dynamics appear to show reduced exploration of the brain state dominated by dorsal–ventral cortical oscillation. Notably, the exploration of the brain state dominated by FPN–SMN/VN oscillation positively correlates with the predominance of manic polarity across the longitudinal course of BD. This pattern suggests that a relative shift of brain dynamics toward a dorsal axis of exteroceptive–associative processing, promoting perception–imagery coupling primarily influenced by interactions with the external environment, may contribute to a longitudinal tendency toward mania. Thus, integrating results from k=6 and k=8 analyses, alterations in brain dynamics converge on a key role of over-tuning of intrinsic brain activity and corresponding phenomenal/behavioural patterns toward the external environment in mania.

In conclusion, CAP analysis with k=8 yielded results consistent with those obtained with k=6 for depression, while importantly complementing them in both depression and mania. Collectively, alterations in brain dynamics appear to converge on opposing neurophenomenal tuning to the external environment in manic-depressive psychopathology.

Limitations

Some limitations should be considered when interpreting the results of this study.

One main limitation is the possible confounding effects of medication. All BD patients in our sample were taking medications, including mood stabilizers, antipsychotics, antidepressants, and benzodiazepines, which could influence the investigated neuroimaging metrics. Following earlier suggestions and standards, we examined the impact of psychotropic medications on our results[21]. Antipsychotics were converted into chlorpromazine dose-equivalents[22], mood stabilizers into lithium dose equivalents[22], antidepressants into imipramine dose-equivalents[22], and benzodiazepines into diazepam dose-equivalents[23]. Medication loads for each class were calculated using the codes 0, 1, 2, and 3 to indicate no medication, and dose-equivalents below, equal to, or above the mean effective daily dose, respectively; the total medication load was defined as the sum of the four classes[24]. The resulting medication dose equivalents and medication load were entered

into correlation analyses with the occurrence rates of CAPs (for both k=6 and k=8) within the BD group. No significant correlations were observed, suggesting that pharmacological treatment is unlikely to directly account for the main findings of this work. See **Supplementary Table 4a** and **Supplementary Table 4b**.

Age differences represent another potential confound. BD patients and HC were not matched for age, with a statistically significant group difference ($F=26.5$, $p<0.001$). Post hoc analyses indicated that both manic and depressed patients were older than HC ($p<0.001$), whereas no age difference was observed between manic and depressed patients ($p=0.99$). Thus, age differences between patients and controls could have influenced the observed differences in brain dynamics. Age was included as a covariate in all group comparisons; while this reduces potential confounding, it may not fully account for age-related effects in patient–control contrasts. Importantly, age cannot explain the differences in brain dynamics observed between manic and depressed states. Overall, these observations indicate that age represents a potential confound for patient–control comparisons, but is unlikely to drive the main findings regarding state-dependent brain dynamics in BD.

The relatively modest sample size may limit statistical power and generalisability of the findings. Nevertheless, the inclusion of both manic and depressive states enhances the clinical relevance and interpretability of the results.

Finally, our study investigated brain dynamics in patients experiencing either the manic or depressive state of BD, which does not allow for within-subject examination of state-dependent changes. Previous work using CAP analysis in BD to investigate amygdala connectivity dynamics reported longitudinal changes in amygdala CAP engagement, supporting the state-dependent nature of CAP reconfigurations across manic and depressive states[25]. Future longitudinal studies should examine whole-brain dynamics in the same patients as they transition between manic and depressive phases to further elucidate these mechanisms.

Future directions

Various theoretical models have been proposed to explain the biological basis of manic-depressive psychopathology. The limbic–cortical dysregulation model posits that hyperactivity in ventral emotion-generating limbic regions, combined with hypoactivity in dorsal emotion-regulating prefrontal areas, leads to emotional dysregulation, manifesting as depression or mania[26-29]. The triple-network model posits that abnormal SN-mediated modulation of switching between DMN and FPN underlies emotional and cognitive disturbances, potentially manifesting as altered saliency processing, internally focused thought (e.g., rumination), and deficits in goal-directed behaviour in depression[30]. The reward-circuit and dopamine-based models propose that altered dopaminergic signalling within mesolimbic circuitry contributes to mood disturbances, with hyper-dopaminergic states and heightened reward processing in mania, and hypo-dopaminergic states and blunted reward processing in depression[31]. Together, these models frame manic-depressive psychopathology in terms of alterations within specific brain regions, circuits, neurotransmitter systems, or canonical networks, largely emphasizing affective dysregulation and, to a lesser extent, cognitive dysfunction, most consistently described in depression. More recently, we delineated a unified model of the pathophysiology of BD, positing that structural alterations in limbic circuitry may alter neurotransmitter signalling and promote a functional reconfiguration of intrinsic brain activity, primarily reflected in an imbalance between SMN and DMN, which ultimately manifests in manic-depressive symptomatology[32-34].

The findings from this work support our previously proposed model and, more importantly, refine and extend it. The observed polarisation of brain dynamics between low-order sensorimotor and high-order associative regions in BD suggests a global distortion of functional brain architecture, producing opposing neurophenomenal tuning to the external environment, with immediate

interaction in mania and detachment in depression. Accordingly, in the context of our theoretical framework, these results shift the focus from localized brain dysfunction to a global and dynamic reconfiguration of brain activity and its associated phenomenal/behavioural patterns in the fundamental organism–environment interaction. This framework may reconcile previously fragmented brain alterations and integrate affective, cognitive, and psychomotor changes, capturing the heterogeneous symptom constellations of BD in line with the classical Kraepelinian model[35], within a unifying perspective of brain alterations and manic-depressive psychopathology.

More specifically, in terms of brain architecture, this framework suggests integrating alterations in limbic and high-order prefrontal/associative regions with fundamental changes in low-order sensorimotor regions (included in the SMN and VN). Previously neglected relative to limbic/emotional and high-order/cognitive systems, alterations in low-order regions and networks have been robustly detected in mania and depression[3, 11-13, 25, 36, 37]. Because these areas serve as the primary interface with the environment, their disruption may reflect a fundamental alteration of organism–environment coupling in BD, which should be considered in the biological models of the disorder.

By relating distortions in the functional brain architecture to psychopathology, this framework may integrate affective and cognitive disturbances with alterations in psychomotricity, while enabling a more detailed hierarchical and phenomenological analysis of manic-depressive symptoms (e.g., distinguishing perception from imagery contributions). By grounding psychopathology in abnormal organism–environment interaction, this approach may help define symptom profiles, identify potential subtypes, and support more precise brain–symptom mapping in BD.

Finally, by relating distortions in the functional brain architecture to their potential drivers, this framework suggests that the observed opposing polarisation of brain dynamics to sensorimotor and associative areas in mania and depression may arise from altered neurotransmitter signalling. This is consistent with prior work showing that dopamine can enhance SMN activity while suppressing DMN activity, whereas serotonin can enhance DMN activity while suppressing SMN activity[15]. By integrating and extending existing limbic- and dopamine-based models, localized alterations in limbic circuitry may modify neurotransmitter signalling, contributing to a global functional reconfiguration of brain activity. These considerations provide mechanistic hypotheses for the pathophysiology of BD.

Taken together, the findings from this work may help refine and integrate current psychopathological and pathophysiological models of BD. The observed polarisation of brain dynamics in mania and depression may represent a key interface between underlying biological drivers and symptom expression. Interpreted within these theoretical models, these findings generate testable hypotheses that could advance mechanistic understanding of the pathophysiology of BD, which in turn may guide future development of biomarkers and therapeutic strategies targeting the disorder itself rather than its symptoms.

References

1. Magioncalda P, Martino M, Conio B, Escelsior A, Piaggio N, Presta A *et al.* Functional connectivity and neuronal variability of resting state activity in bipolar disorder--reduction and decoupling in anterior cortical midline structures. *Human brain mapping* 2015; **36**(2): 666–682.
2. Magioncalda P, Martino M, Conio B, Piaggio N, Teodorescu R, Escelsior A *et al.* Patterns of microstructural white matter abnormalities and their impact on cognitive dysfunction in the various phases of type I bipolar disorder. *J Affect Disord* 2016; **193:** 39–50.
3. Martino M, Magioncalda P, Huang Z, Conio B, Piaggio N, Duncan NW *et al.* Contrasting variability patterns in the default mode and sensorimotor networks balance in bipolar depression and mania. *Proceedings of the National Academy of Sciences of the United States of America* 2016; **113**(17): 4824–4829.
4. Martino M, Magioncalda P, Saiote C, Conio B, Escelsior A, Rocchi G *et al.* Abnormal functional-structural cingulum connectivity in mania: combined functional magnetic resonance imaging-diffusion tensor imaging investigation in different phases of bipolar disorder. *Acta Psychiatr Scand* 2016; **134**(4): 339–349.
5. Martino M. A working model on large-scale spatio-temporal organization of brain functioning and its implications for bipolar disorder. PhD thesis, University of Genoa, Genoa (Italy), 2018.
6. Magioncalda P, Martino M, Tardito S, Sterlini B, Conio B, Marozzi V *et al.* White matter microstructure alterations correlate with terminally differentiated CD8+ effector T cell depletion in the peripheral blood in mania: Combined DTI and immunological investigation in the different phases of bipolar disorder. *Brain, behavior, and immunity* 2018; **73:** 192–204.
7. Northoff G, Magioncalda P, Martino M, Lee HC, Tseng YC, Lane T. Too Fast or Too Slow? Time and Neuronal Variability in Bipolar Disorder-A Combined Theoretical and Empirical Investigation. *Schizophr Bull* 2018; **44**(1): 54–64.
8. Piaggio N, Schiavi S, Martino M, Bommarito G, Inglese M, Magioncalda P. Exploring mania-associated white matter injury by comparison with multiple sclerosis: a diffusion tensor imaging study. *Psychiatry Res Neuroimaging* 2018; **281:** 78–84.
9. Magioncalda P. Different patterns of white matter and immunological alterations in the various phases of bipolar disorder. PhD thesis, University of Genoa, Genoa (Italy), 2019.
10. Conio B, Magioncalda P, Martino M, Tumati S, Capobianco L, Escelsior A *et al.* Opposing patterns of neuronal variability in the sensorimotor network mediate cyclothymic and depressive temperaments. *Hum Brain Mapp* 2019; **40**(4): 1344–1352.
11. Zhang J, Magioncalda P, Huang Z, Tan Z, Hu X, Hu Z *et al.* Altered Global Signal Topography and Its Different Regional Localization in Motor Cortex and Hippocampus in Mania and Depression. *Schizophr Bull* 2019; **45**(4): 902–910.
12. Martino M, Magioncalda P, Conio B, Capobianco L, Russo D, Adavastro G *et al.* Abnormal Functional Relationship of Sensorimotor Network With Neurotransmitter-Related Nuclei via Subcortical-Cortical Loops in Manic and Depressive Phases of Bipolar Disorder. *Schizophr Bull* 2020; **46**(1): 163–174.
13. Russo D, Martino M, Magioncalda P, Inglese M, Amore M, Northoff G. Opposing Changes in the Functional Architecture of Large-Scale Networks in Bipolar Mania and Depression. *Schizophr Bull* 2020; **46**(4): 971–980.
14. Magioncalda P, Martino M, Conio B, Lee HC, Ku HL, Chen CJ *et al.* Intrinsic brain activity of subcortical-cortical sensorimotor system and psychomotor alterations in schizophrenia and bipolar disorder: A preliminary study. *Schizophr Res* 2020; **218:** 157–165.

15. Conio B, Martino M, Magioncalda P, Escelsior A, Inglese M, Amore M *et al.* Opposite effects of dopamine and serotonin on resting-state networks: review and implications for psychiatric disorders. *Mol Psychiatry* 2020; **25**(1)**:** 82–93.
16. Conio B. Neurotransmitters and resting state networks: clinical implication for major psychiatric disorder. PhD thesis, University of Genoa, Genoa (Italy), 2021.
17. Escelsior A, Sterlini B, Tardito S, Altosole T, Magioncalda P, Martino M *et al.* Evidence of alterations of Beta-endorphin levels and Mu-opioid receptor gene expression in bipolar disorder. *Psychiatry Res* 2022; **316:** 114787.
18. Chen HT, Martino M, Dabiri E, Tamara FR, Sibiya L, Conio B *et al.* Biological correlates of temperament: systematic reviews, empirical studies, and a conceptual framework linking neurotransmitter signaling, intrinsic brain activity, and the hyperthymic-depressive spectrum. *Mol Psychiatry* 2025; **30**(12)**:** 5880–5888.
19. Yeo BT, Krienen FM, Sepulcre J, Sabuncu MR, Lashkari D, Hollinshead M *et al.* The organization of the human cerebral cortex estimated by intrinsic functional connectivity. *J Neurophysiol* 2011; **106**(3)**:** 1125–1165.
20. Gutierrez-Barragan D, Ramirez JSB, Panzeri S, Xu T, Gozzi A. Evolutionarily conserved fMRI network dynamics in the mouse, macaque, and human brain. *Nat Commun* 2024; **15**(1)**:** 8518.
21. Phillips ML, Travis MJ, Fagiolini A, Kupfer DJ. Medication effects in neuroimaging studies of bipolar disorder. *The American journal of psychiatry* 2008; **165**(3)**:** 313–320.
22. Baldessarini RJ. *Chemotherapy in Psychiatry. Pharmacologic Basis of Treatments of Major Mental Illness*. 3rd edn. Springer: New York, 2013.
23. Arana GW RJ. *Handbook of Psychiatric Drug Therapy*. Lippincott, Williams and Wilkins: Philadelphia, PA, 2000.
24. Davis JM, Chen N. Dose response and dose equivalence of antipsychotics. *Journal of clinical psychopharmacology* 2004; **24**(2)**:** 192–208.
25. Rey G, Bolton TAW, Gaviria J, Piguet C, Preti MG, Favre S *et al.* Dynamics of amygdala connectivity in bipolar disorders: a longitudinal study across mood states. *Neuropsychopharmacology* 2021; **46**(9)**:** 1693–1701.
26. Mayberg HS. Limbic-cortical dysregulation: a proposed model of depression. *J Neuropsychiatry Clin Neurosci* 1997; **9**(3)**:** 471–481.
27. Phillips ML, Swartz HA. A critical appraisal of neuroimaging studies of bipolar disorder: toward a new conceptualization of underlying neural circuitry and a road map for future research. *Am J Psychiatry* 2014; **171**(8)**:** 829–843.
28. Strakowski SM, Adler CM, Almeida J, Altshuler LL, Blumberg HP, Chang KD *et al.* The functional neuroanatomy of bipolar disorder: a consensus model. *Bipolar Disord* 2012; **14**(4)**:** 313–325.
29. Savitz J, Drevets WC. Bipolar and major depressive disorder: neuroimaging the developmental-degenerative divide. *Neurosci Biobehav Rev* 2009; **33**(5)**:** 699–771.
30. Menon V. Large-scale brain networks and psychopathology: a unifying triple network model. *Trends Cogn Sci* 2011; **15**(10)**:** 483–506.
31. Ashok AH, Marques TR, Jauhar S, Nour MM, Goodwin GM, Young AH *et al.* The dopamine hypothesis of bipolar affective disorder: the state of the art and implications for treatment. *Mol Psychiatry* 2017; **22**(5)**:** 666–679.
32. Magioncalda P, Martino M. A unified model of the pathophysiology of bipolar disorder. *Mol Psychiatry* 2022; **27**(1)**:** 202–211.
33. Martino M, Magioncalda P. Tracing the psychopathology of bipolar disorder to the functional architecture of intrinsic brain activity and its neurotransmitter modulation: a three-dimensional model. *Mol Psychiatry* 2022; **27**(2)**:** 793–802.
34. Martino M, Magioncalda P. A three-dimensional model of neural activity and phenomenal-behavioral patterns. *Mol Psychiatry* 2024; **29**(3)**:** 639–652.

35. Kraepelin E. Clinical psychiatry. Macmillan: London, 1902.
36. Mavar S, Lee YS, Baranova E, Duncan NW, Magioncalda P, Martino M. A working model linking the psychopathology and pathophysiology of major depressive disorder - an umbrella review of neuroimaging studies and a conceptual framework. *Mol Psychiatry* 2025; **30**(12)**:** 6007–6019.
37. Zhang Y, Huang CC, Zhao J, Liu Y, Xia M, Wang X. Dysfunction in sensorimotor and default mode networks in major depressive disorder with insights from global brain connectivity. *Nat Mental Health* 2024; **2:** 1371–1381.

Supplementary Table 1. Subject demographic and clinical information

	HC	MANIA	DEPRESSION
Sample size *n*	73	34	35
Age *mean (SD)*	33.1 (10.6)	45.8 (10.3)	45.9 (10.2)
Female *n (%)*	45 (61.6)	22 (64.7)	20 (57.1)
Male *n (%)*	28 (38.4)	12 (35.3)	15 (42.9)
YMRS total score *mean (SD)*	-	18.2 (5.2)	4.0 (2.9)
HAM-D total score *mean (SD)*	-	7.2 (5.6)	21.4 (3.9)
Current episode duration (months) *mean (SD)*	-	3.6 (4.6)	20.2 (61.2)
Illness duration *mean (SD)*	-	17.6 (13.5)	17.4 (12.2)
Number of total episodes *mean (SD)*	-	9.6 (10.4)	8.1 (8.2)
Number of previous manic episodes *mean (SD)*	-	2.3 (3.8)	0.3 (0.8)
Number of previous hypomanic episodes *mean (SD)*	-	1.8 (4.3)	1.8 (4.0)
Number of previous depressive episodes *mean (SD)*	-	2.4 (4.6)	2.5 (4.1)
Number of previous mixed episodes *mean (SD)*	-	2.1 (4.1)	3.6 (6.8)
Manic-depressive polarity *mean (SD)*	-	2.2 (3.0)	1.0 (0.4)
Mood stabilizers *n (%)*	-	29 (85.3)	28 (80)
Antidepressants *n (%)*	-	4 (11.8)	20 (57.1)
Antipsychotics *n (%)*	-	22 (64.7)	26 (74.3)
Benzodiazepines *n (%)*	-	16 (47.1)	22 (62.9)

Abbreviations: HC, healthy controls; YMRS, Young Mania Rating Scale; HAM-D, Hamilton Depression Rating Scale; SD, standard deviation.

Supplementary Table 2. Differences in CAP occurrence rates between healthy controls, mania, and depression (k=8)

CAP	HC *mean (SD)*	M *mean (SD)*	D *mean (SD)*	ANOVA *F (p)*	M vs. HC *p (p*)*	D vs. HC *p (p*)*	M vs. D *p (p*)*
**CAP 1**	0.124 (0.05)	0.143 (0.05)	0.131 (0.04)	1.33 (0.26)	M>HC 0.11	D>HC 0.53	M>D 0.39
**CAP 2**	0.131 (0.04)	0.127 (0.05)	0.159 (0.04)	**4.97 (0.008)**	M<HC 0.73	**D>HC 0.010 (0.029)**	**D>M 0.005 (0.015)**
**CAP 3**	0.129 (0.05)	0.143 (0.05)	0.131 (0.05)	0.66 (0.51)	M>HC 0.27	D>HC 0.85	M>D 0.38
**CAP 4**	0.133 (0.04)	0.135 (0.05)	0.164 (0.04)	**5.89 (0.004)**	M>HC 0.89	**D>HC 0.002 (0.007)**	**D>M 0.005 (0.015)**
**CAP 5**	0.132 (0.05)	0.100 (0.05)	0.106 (0.05)	**4.01 (0.02)**	**M<HC 0.010 (0.029)**	**D<HC 0.034** (0.10)	M<D 0.63
**CAP 6**	0.106 (0.039	0.120 (0.04)	0.101 (0.04)	1.93 (0.14)	M>HC 0.14	D<HC 0.65	M>D 0.063
**CAP 7**	0.138 (0.05)	0.116 (0.05)	0.109 (0.06)	**3.15 (0.046)**	M<HC 0.077	**D<HC 0.018** (0.055)	D<M 0.57
**CAP 8**	0.107 (0.03)	0.116 (0.04)	0.099 (0.04)	1.74 (0.17)	M>HC 0.32	D<HC 0.34	M>D 0.064

Differences in occurrence rates of co-activation patterns (CAPs) between healthy controls (HC), mania (M), and depression (D) were assessed using ANOVA, with age and sex as covariates, followed by post-hoc comparisons (values are adjusted for age and sex). p = uncorrected post-hoc contrasts; p* = Bonferroni-corrected post-hoc contrasts. Values in bold indicate significant results.

Supplementary Table 3a. Relationship of CAP occurrence rates with clinical features (k=6)

	**CAP 1** *rho (p)*	**CAP 2** *rho (p)*	**CAP 3** *rho (p)*	**CAP 4** *rho (p)*	**CAP 5** *rho (p)*	**CAP 6** *rho (p)*
YMRS/HAM-D RATIO	0.10 (0.43)	0.12 (0.31)	-0.21 (0.09)	-0.01 (0.92)	**-0.36 (0.002)**	**0.41 (<0.001)**
YMRS TOTAL SCORE	0.10 (0.43)	0.16 (0.18)	-0.22 (0.06)	0.04 (0.71)	**-0.37 (0.001)**	**0.34 (0.004)**
HAM-D TOTAL SCORE	-0.05 (0.70)	-0.09 (0.46)	0.14 (0.25)	0.11 (0.38)	0.21 (0.07)	**-0.38 (0.001)**
Current episode duration	0.12 (0.33)	0.01 (0.98)	-0.04 (0.76)	0.01 (0.95)	-0.02 (0.85)	-0.02 (0.85)
Illness duration	0.06 (0.58)	0.07 (0.51)	-0.14 (0.23)	0.11 (0.34)	-0.09 (0.42)	-0.05 (0.63)
Number of total episodes	0.05 (0.69)	0.08 (0.50)	-0.05 (0.69)	-0.05 (0.68)	-0.03 (0.78)	0.02 (0.89)
Manic-depressive polarity	**0.32 (0.008)**	-0.16 (0.18)	-0.09 (0.45)	-0.09 (0.45)	-0.22 (0.08)	**0.35 (0.003)**

Supplementary Table 3b. Relationship of CAP occurrence rates with clinical features (k=8)

	**CAP 1** *rho (p)*	**CAP 2** *rho (p)*	**CAP 3** *rho (p)*	**CAP 4** *rho (p)*	**CAP 5** *rho (p)*	**CAP 6** *rho (p)*	**CAP 7** *rho (p)*	**CAP 8** *rho (p)*
YMRS/HAM-D RATIO	*0.23 (0.05)*	*-0.31 (0.009)*	*0.25 (0.04)*	*-0.28 (0.02)*	-0.07 (0.56)	0.04 (0.73)	0.01 (0.94)	0.12 (0.31)
YMRS TOTAL SCORE	0.18 (0.13)	*-0.34 (0.004)*	0.18 (0.15)	*-0.30 (0.01)*	-0.01 (0.95)	0.06 (0.62)	0.08 (0.49)	0.07 (0.55)
HAM-D TOTAL SCORE	-0.18 (0.15)	0.21 (0.08)	*-0.23 (0.05)*	0.22 (0.06)	0.15 (0.20)	-0.11 (0.38)	-0.07 (0.57)	-0.20 (0.11)
Current episode duration	-0.03 (0.82)	0.02 (0.86)	-0.09 (0.45)	0.02 (0.84)	0.02 (0.85)	0.14 (0.24)	-0.1 (0.91)	-0.03 (0.82)
Illness duration	-0.03 (0.83)	-0.08 (0.53)	-0.01 (0.99)	-0.12 (0.31)	0.07 (0.57)	-0.02 (0.90)	0.06 (0.64)	0.12 (0.31)
Number of total episodes	0.07 (0.55)	-0.09 (0.47)	0.04 (0.74)	-0.04 (0.73)	-0.09 (0.47)	0.10 (0.44)	-0.05 (0.69)	0.13 (0.29)
Manic-depressive polarity	**0.38 (0.001)**	-0.23 (0.06)	**0.36 (0.003)**	-0.18 (0.13)	-0.10 (0.43)	-0.04 (0.77)	-0.17 (0.17)	-0.03 (0.78)

The relationship between occurrence rates of co-activation patterns (CAPs) and manic-depressive symptomatology—Young Mania Rating Scale (YMRS) and Hamilton Depression Rating Scale (HAM-D) scores—as well as other clinical variables, was assessed using Spearman correlation analysis. Italicized values indicate significant results; bold values indicate significant results surviving Bonferroni correction.

Supplementary Table 4a. Relationship of CAP occurrence rates with medications (k=6)

	**CAP 1** *rho (p)*	**CAP 2** *rho (p)*	**CAP 3** *rho (p)*	**CAP 4** *rho (p)*	**CAP 5** *rho (p)*	**CAP 6** *rho (p)*
Lithium dose equivalents	-0.14 (0.24)	0.11 (0.36)	-0.08 (0.47)	0.06 (0.58)	-0.10 (0.38)	0.12 (0.31)
Chlorpromazine dose equivalents	0.01 (0.93)	-0.04 (0.74)	0.01 (0.98)	0.01 (0.92)	0.06 (0.61)	-0.06 (0.58)
Imipramine dose equivalents	-0.14 (0.23)	0.05 (0.63)	0.02 (0.85)	0.11 (0.36)	0.07 (0.55)	-0.22 (0.06)
Diazepam dose equivalents	0.06 (0.57)	0.03 (0.779	-0.07 (0.54)	-0.09 (0.45)	-0.01 (0.88)	0.11 (0.35)
Medication load	0.03 (0.79)	-0.03 (0.75)	0.01 (0.93)	-0.10 (0.40)	0.06 (0.59)	0.01 (0.94)

Supplementary Table 4b. Relationship of CAP occurrence rates with medications (k=8)

	**CAP 1** *rho (p)*	**CAP 2** *rho (p)*	**CAP 3** *rho (p)*	**CAP 4** *rho (p)*	**CAP 5** *rho (p)*	**CAP 6** *rho (p)*	**CAP 7** *rho (p)*	**CAP 8** *rho (p)*
Lithium dose equivalents	0.01 (0.91)	-0.06 (0.60)	-0.04 (0.77)	-0.01 (0.99)	0.12 (0.33)	-0.08 (0.54)	0.04 (0.76)	-0.01 (0.90)
Chlorpromazine dose equivalents	0.09 (0.44)	-0.11 (0.39)	0.13 (0.28)	-0.15 (0.30)	-0.09 (0.45)	0.11 (0.36)	-0.08 (0.50)	0.12 (0.32)
Imipramine dose equivalents	-0.20 (0.09)	0.03 (0.81)	-0.12 (0.32)	0.02 (0.87)	0.19 (0.12)	0.04 (0.73)	0.13 (0.29)	-0.11 (0.35)
Diazepam dose equivalents	0.18 (0.15)	-0.03 (0.83)	0.16 (0.18)	-0.09 (0.44)	-0.14 (0.24)	-0.04 (0.75)	-0.03 (0.83)	-0.02 (0.89)
Medication load	-0.01 (0.97)	-0.01 (0.98)	0.01 (0.93)	-0.08 (0.51)	0.04 (0.74)	0.01 (0.92)	0.05 (0.70)	-0.04 (0.75)

The relationship between occurrence rates of co-activation patterns (CAPs) and medications was assessed using Spearman correlation analysis.

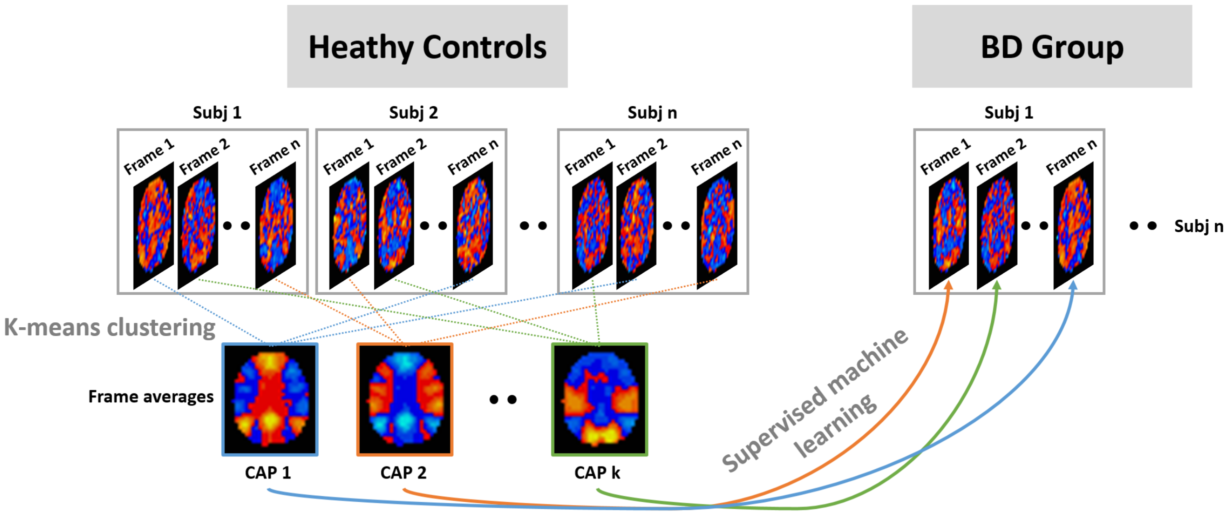

Supplementary Figure 1. CAP analysis
Distinct co-activation patterns (CAPs) were identified using a k-means clustering algorithm in healthy controls. For each cluster, a representative CAP map was generated. These maps were then applied to data from patients with bipolar disorder (BD) to identify similar patterns.

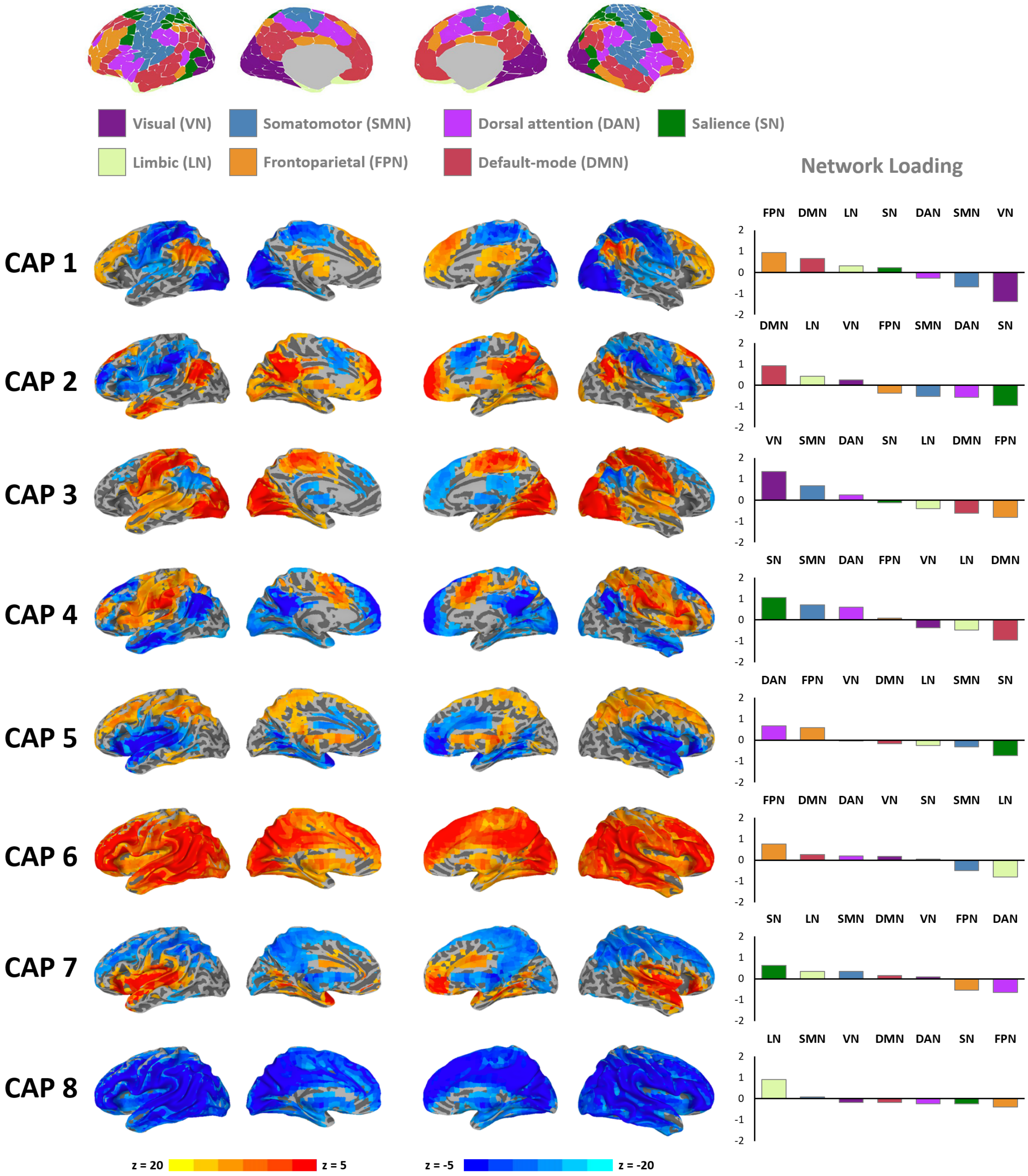

Supplementary Figure 2. Spatial maps and network contributions of CAPs (k=8)
Co-activation pattern (CAP) maps reveal distinct spatial configurations of coordinated brain activity. The accompanying bar plots quantify the involvement of key brain networks—visual (VN), somatomotor (SMN), salience (SN), dorsal attention (DAN), limbic (LN), frontoparietal (FPN), and default-mode (DMN)—in each CAP.

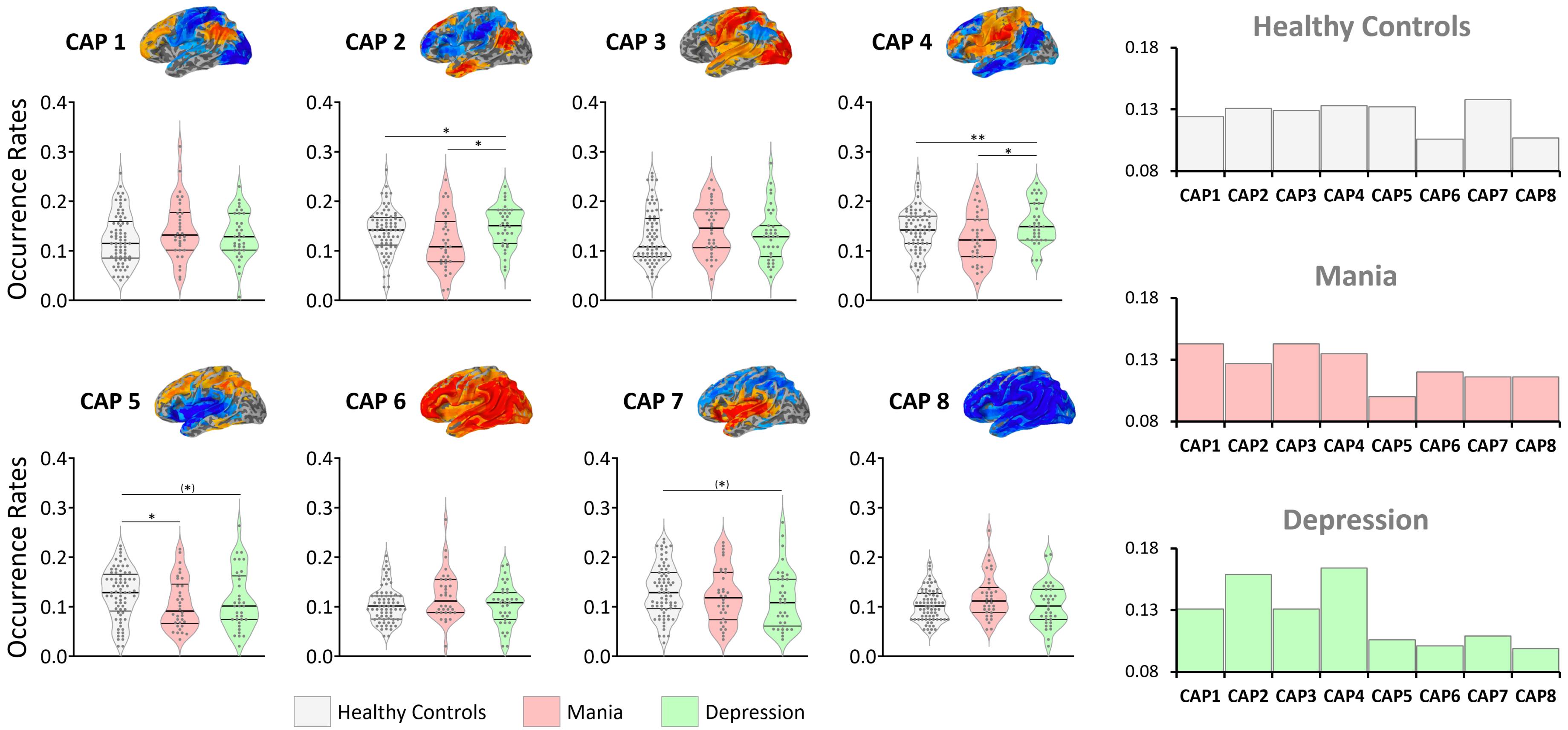

Supplementary Figure 3. Differences in CAP occurrence rates between healthy controls, mania, and depression (k=8)
Differences in occurrence rates of co-activation patterns (CAPs) between healthy controls, mania, and depression were assessed using ANOVA, with age and sex as covariates, followed by post-hoc comparisons. (*) $p<0.05$ (uncorrected); * $p<0.05$ (Bonferroni-corrected); ** $p<0.01$ (Bonferroni-corrected). Bar plots illustrate the average occurrence of each CAP, adjusted for age and sex.
